# Predictors of Sleep Duration and Sleep Disturbance in Children of a Culturally Diverse Region in North-Eastern Greece

**DOI:** 10.3389/fped.2020.00023

**Published:** 2020-02-12

**Authors:** Evangelia Nena, Dimitrios Cassimos, Athanasios Kaditis, Maria Kourantzi, Georgia Trakada, Nikolaos-Tiberio Economou, Pantelis T. Nikolaidis, Thomas Rosemann, Beat Knechtle, Paschalis Steiropoulos, Angelos Tsalkidis

**Affiliations:** ^1^Laboratory of Hygiene and Environmental Protection, Medical School, Democritus University of Thrace, Alexandroupolis, Greece; ^2^Department of Paediatrics, Medical School, Democritus University of Thrace, Alexandroupolis, Greece; ^3^Pediatric Pulmonology Unit, First Dept of Paediatrics, University of Athens School of Medicine, Aghia Sophia Children's Hospital, Athens, Greece; ^4^Division of Pulmonology, Department of Clinical Therapeutics, National and Kapodistrian University of Athens School of Medicine, Alexandra Hospital, Athens, Greece; ^5^Exercise Physiology Laboratory, Nikaia, Greece; ^6^School of Health and Caring Sciences, University of West Attica, Athens, Greece; ^7^Institute of Primary Care, University of Zurich, Zurich, Switzerland; ^8^Medbase St. Gallen Am Vadianplatz, St. Gallen, Switzerland; ^9^Sleep Laboratory, Department of Pneumonology, Medical School, Democritus University of Thrace, Alexandroupolis, Greece

**Keywords:** children, cultural diversity, sleep disorders, socio-demographic factors, Greece

## Abstract

**Background Aim:** The aim of this study was to examine the sleep characteristics of children and explore associations with various socio-demographic factors in an area of Greece characterized by cultural diversity.

**Methods:** A questionnaire about children's sleep habits had been distributed to parents of children who visited the pediatric outpatient clinic of University General Hospital of Alexandroupolis for a medical examination and to get a health certificate for participation in sports activities. Children with chronic health conditions were excluded.

**Results:** In the study, 449 children (27.1% belonging to minorities) were included, aged 7.6 ± 2.9 years. Most of them (81.7%) slept after 10 p.m., with a mean nocturnal sleep duration of 9.4 ± 1.2 h. The most commonly reported disturbance was snoring (26.2%). Age and high educational level of the mother were both important determinants for sleeping late [OR 1.139 (1.033–1.255); *p* = 0.009 and OR 1.086 (1.004–1.175); *p* = 0.040, respectively]. The latter was also associated with an absence of any reported sleep disorder in children [OR 0.934 (0.877–0.994); *p* = 0.031]. A longer sleep duration was reported among Roma children (*p* = 0.022), which was more overt in girls (mean sleep duration 10.4 ± 1.6 h). In the Roma group also, the prevalence of sleep disorders was higher.

**Conclusion:** Age progression and maternal educational level, along with cultural background, seem to be correlated with variations in the sleep characteristics of children in a culturally diverse population in Greece.

## Introduction

Sleep disturbances may have a negative impact on the physical and mental health of children. Inadequate sleep duration in children has been associated with weight gain, impaired cognitive skills, poor academic performance, and the presence of mood disorders (1–3). Sleep duration in childhood is affected not only by biological and environmental factors but also by socio-demographic and behavioral factors and varies significantly between different countries (4–7). Differences in cultural characteristics may also play a significant role in sleep duration, especially with reference to time of sleep initiation. Children from northern European countries sleep longer than children from southern Europe (8), while in some ethnic minority groups, the prevalence of short sleep is higher (9). In the United States of America, the prevalence of serious sleep problems is higher in children living in neighborhoods with poor socioeconomic conditions compared to children from neighborhoods of a favorable socioeconomic environment (10). Children of a deprived background in England tend to sleep later at night (11).

The region of Thrace in Northeastern Greece comprises three cities and numerous towns and villages, with a local population characterized by diversity, with religious and cultural differences between different groups (12). Therefore, it could be an ideal setting for evaluating sleep characteristics and comparing the effects of different socio-demographic factors on them. The aim of the current study was to assess the sleep characteristics and disturbances of children living in the above area and examine their associations with socio-demographic factors.

## Methods

### Ethical Approval

The study was approved by the Medical School, Democritus University of Thrace and the Institutional Review Board of the University General Hospital of Alexandroupolis, Greece (1/7-10-2011).

### Participants

The study was conducted in two phases in 2009-10 and 2010-11 between October and February, in order to eliminate the effect of climate conditions (i.e., daylight and high temperatures) and the variations of sleep during summer vacations.

All parents escorting children at the outpatient pediatric clinic of the Alexandroupolis University General Hospital were consecutively asked to participate in an interview with the use of a structured questionnaire. The only inclusion criteria were: children referred for health certification. Children with acute or chronic health problems were excluded from the study. The investigator asked questions about the sleep habits of the child, as well as to gather indirect information about the socioeconomic status of the family, namely the educational level of both parents and the living conditions at home. All procedures performed in the study were in accordance with the ethical standards of the institutional ethics committee, which approved its conduct, and with the standards of the Helsinki declaration (1964) and its later amendments. Informed consent was obtained from the parents of all individual participants included in the study.

### Definitions

All sleep disturbances were subjectively assessed based on the parents' answers to the delivered questionnaire. No objective assessment was conducted. The following sleep disturbances were explored, based on the presence of at least one of the following reported conditions: (i) insomnia, defined as the presence of one of the following: difficulty in falling asleep and/or staying asleep, including bedtime refusal and resistance and night-time awakenings requiring parental intervention (13); (ii) awakenings during at least 2 nights per week, with crying and asking for parents' company; (iii) snoring at least half of the sleep time; (iv) observed apnea during sleep; (v) periodic limb movements (these were recorded if the parent had observed repeated movements of the legs during the child's sleep); (vi) excessive daytime sleepiness (assessed as follows: the parent had difficulty waking up the child in the morning; the child was feeling fatigued after waking up; the child was sleepy during daytime as observed by the parent or as the school teacher reported to the parent).

### Data Analysis

Sample size calculation: According to the child population size in the area, based on data from the Hellenic Statistical Authority (www.statistics.gr), the ideal sample size in order to ensure a 95% confidence level (*p* = 0.05) and a 5% margin of error would be 323. Normality was assessed by the Kolmogorov-Smirnov test, and parametric statistics were applied to normally distributed data. For differences in continuous variables, the Student *t*-test or the Analysis of Variance (ANOVA) was applied, while for categorical variables, the chi-squared test was used. Data are expressed as mean ± SD unless otherwise indicated. For describing time events (i.e., the time when children are put to sleep or the time when they wake up), the following form was applied: Mean estimated time (expressed as HH:MM) ± SD (expressed in minutes). Multivariable logistic regression analysis was used to calculate odds ratio and the corresponding 95% confidence intervals (95% CI) for the following outcomes: sleep duration, going to bed after 10 p.m., or presence of any sleep disturbance. Explanatory variables included: age, gender, cultural-religious background (i.e., natively-born Greek of the Muslim minority or Roma), socio-demographic factors [years of education of each parent, total number of persons in the house (> 4 vs. ≤4), and total number of persons in the child's bedroom (>1 vs. 1)], child-raising practices (parent who is mainly responsible for raising the child, parent who is responsible for putting the child to bed). IBM SPSS Statistics for Windows, Version 19.0 (Armonk, NY: IBM Corp.) was used for data analysis. Statistical significance was set at a *p* < 0.05.

## Results

### General Characteristics of Participants

Of the 532 initially approached during the study period, 449 agreed to participate (participation rate 84.4%). The mean age ± standard deviation of participating children was 7.6 ± 2.9 years (range 3.5–14 years). The majority were males (59.5%), with no difference observed between genders. More specifically: the female age was 7.6 ± 2.9 years, while the male age was 7.5 ± 2.9 years (*p* = 0.777). Regarding cultural background, 27.1% belonged to minorities, i.e., 16% were natively born Greeks of the Muslim religion and 11.1% were Roma. No significant difference in age between groups was observed (*p* = 0.429).

Regarding the educational level of parents: mean years of education were 9.7 ± 4.9 for the mothers and 9.4 ± 4.8 for the fathers. In the vast majority, the mothers were responsible for raising the children, either alone (47.4%) or with the father (42.1%). In line with this, the mother was usually responsible for putting the child to bed, either alone (50.8%) or together with the father (10.7%). In most cases, families included four persons (45.7%), and children shared their room with another sibling (43.2%). However, in the Roma group, it was reported that ≥2 other persons (siblings, parents, or grandparents) shared the same room with the children.

### Sleep Duration and Time of Going to Bed

The mean nocturnal sleep duration was 9.4 ± 1.2 h. Only 36.1% of all children were napping during the day for 2 ± 0.7 h. Both nocturnal sleep duration and napping duration were negatively correlated with age (*p* < 0.001 in both cases). A statistically significant difference was revealed between genders regarding duration of sleep, with a longer duration observed in females (9.6 ± 1.2 vs. 9.3 ± 1.1 h; *p* = 0.027). A regression model that was formed to assess the effect of the different parameters revealed that older age was associated with a lower OR for sleeping more than 9 h (OR: 0.871; 95% CI: 0.802–0.947, *p* = 0.001). None of the other parameters entered in this regression model, namely gender, educational level of the mother, educational level of the father, person responsible for raising the child, person responsible for putting the child to sleep, number of persons in the house, and number of persons in the same room with the child, could predict longer sleep duration (Table 1).

**Table 1:** Association between various socio-demographic factors and the following outcomes: sleep duration >9 h, going to bed after 10 p.m., and the presence of sleep disturbance (binary logistic regression analysis).

	**Sleep duration > 9 h**		**Going to bed after 10 p.m**.		**Presence of any sleep disturbance**	
	**OR (95% CIs)**	***P***	**OR (95% CIs)**	***P***	**OR (95% CIs)**	***P***
Age	0.871 (0.802–0.947)	0.001	1.139 (1.033–1.255)	0.009	0.969 (0.903–1.039)	0.376
Gender (females vs. males)	1.555 (0.957–2.526)	0.075	0.568 (0.348–0.927)	0.568	0.799 (0.544–1.173)	0.253
Years of education of mother	0.959 (0.889–1.034)	0.271	1.086 (1.004–1.175)	0.040	0.934 (0.877–0.994)	0.031
Years of education of father	1.029 (0.952–1.111)	0.474	0.959 (0.883–1.042)	0.322	1.052 (0.987–1.122)	0.120
Responsible for raising the child (others vs. mother)	1.126 (0.682–1.859)	0.643	0.915 (0.537–1.559)	0.915	0.833 (0.554–1.252)	0.380
Responsible for putting the child to sleep (others vs. mother)	0.744 (0.440–1.256)	0.268	1.014 (0.583–1.763)	0.961	0.807 (0.528–1.234)	0.323
Number of persons in house	0.936 (0.746–1.175)	0.570	1.002 (0.778–1.291)	0.986	0.916 (0.756–1.109)	0.368
Number of persons sharing the same room	1.032 (0.777–1.370)	0.829	1.116 (0.826–1.506)	0.475	1.082 (0.859–1.362)	0.503

The average time of going to bed was at 22:20 ± 1 h, while the average time of awakening was 07:50 ± 1 h. Most children (81.7%) were put to bed after 10 p.m. Girls were put to bed earlier than boys (sleep time: 22:10 ± 56 min vs. 22:25 ± 59 min; *p* = 0.006). Nevertheless, awakening time did not differ between girls and boys (07:48 ± 57 vs. 07:48 ± 58 min; *p* = 0.888). In total, the average time of sleep initiation was at 22:20 ± 1 h, while the average time of awakening was 07:50 ± 1 h. Children, who were put to bed before 22:00 p.m. were significantly younger in comparison to those put to bed after 22:00 p.m. (6.8 ± 2.4 vs. 7.7 ± 2.9 y, *p* = 0.004), while their sleep duration was longer (10.5 ± 1 vs. 9.2 ± 1.1 h, *p* < 0.001). Logistic regression analysis indicated that older age and a better educational level of the mother were associated with a higher probability of sleeping <9 h (Table 1).

As a second step, a comparison between culturally different groups regarding night-time sleep duration was performed, revealing a significant intergroup difference, with Roma children having a longer sleep duration (9.8 ± 1.8 h, *p* = 0.022).

The time when children were put to bed, however, did not differ between these groups (*p* = 0.149). In all groups, the mean sleep time was after 22:00 p.m. There were no significant gender variations regarding sleep duration and sleep and awake time in the different cultural groups. Only in the Roma group was a significantly higher sleep duration observed in girls (10.4 ± 1.6 h) vs. boys (9.3 ± 1.8 h) (*p* = 0.024), as well as a marginally non-significant difference in sleep start time according to sex (21:57 ± 1 h 19 min in girls vs. 22:40 ± 1 h 6 min in boys, *p* = 0.051). No other differences were observed.

### Prevalence of Sleep Disturbances and Sleep Quality

A non-neglectable prevalence (41%) of at least one sleep disturbance was demonstrated in the whole study group, which was even higher in the Roma group (64%). The most commonly reported sleep disturbances were: snoring for more than half of the sleep time (26.1%), periodic limb movement (14.1%), followed by insomnia (5.8%) and excessive daytime sleepiness in 3.8% of participants, which in, the Roma children, rose to 14%.

Children's gender was not associated with the presence of sleep disorder (*p* = 0.284). In contrast, age was an important factor. More specifically, children with observed sleep disturbances were younger (7.2 ± 2.8 years) in comparison to those with no sleep disturbance (7.8 ± 2.9 years, *p* = 0.027). Regression analysis revealed that children of better-educated mothers had a lower likelihood of sleep disturbances [OR: 0.934 (95% CI: 0.877–0.994), *p* = 0.031], while no other parameters were associated (Table 1).

Further analysis revealed that children with reported symptoms indicative of insomnia (i.e., difficulty in starting sleep, frequent awakenings, etc.) lived in houses with significantly more inhabitants (5 ± 2 vs. 4 ± 1; *p* = 0.028). On the other hand, the number of persons sharing the same room did not differ significantly (*p* = 0.135). Daytime somnolence was observed in children of lower socio-economic status, as expressed by a significantly lower number of educational years of either the mother (6.2 ± 6.9 vs. 9.8 ± 4.8, *p* = 0.043) or the father (4.2 ± 5.5 vs. 9.6 ± 4.7, *p* = 0.001). An additional finding was that children with somnolence slept in the same room with a significantly higher number of other persons (3 ± 1.3 vs. 2 ± 1.1; *p* = 0.030).

Most parents (59%) characterized the sleep quality of their child as good, without awakenings or sleep disturbances. Binary logistic regression analysis was applied using a model that examined the impact of the following social parameters on reported sleep quality (characterized as either good or bad): (a) individual responsible for raising the child, (b) individual responsible for putting the child to sleep, (c) number of persons in the house, and (d) number of persons in the same room with the child. The analysis revealed that none of the above-mentioned factors significantly affected the reported sleep quality (*p*-values, respectively: 0.265, 0.854, 0.441, and 0.637; overall statistics: *p* = 0.876).

## Discussion

Our study, conducted in a representative sample of children of diverse backgrounds living in a region of NE Greece, showed that the mean sleep duration was 9.4 h, which was shorter than that reported in other European countries, ranging between 9.5 (Estonia) and 11.2 (Belgium) hours (8). Sleep duration was related to age and gender. The majority of children were put to bed after 10:00 p.m., even on school days. As expected, and in accordance with previous studies, age progression was associated with a reduction in sleep duration and with a later sleep start time. The younger children were more often put to bed before 10:00 p.m., while older children had a higher probability of sleeping <9 h.

In a study conducted in the Netherlands comprising 1,507 children, the mean bedtime was at 8 p.m. (14). An older study comprising over 40,000 children aged 11–16 years from 11 European countries reported that children in Spain (a country similar to Greece in terms of climatic conditions, i.e., temperature, sunlight, and number of sunny days) slept at 10:30 p.m., while in central European countries like Switzerland and Hungary, sleep was initiated at 9:30 p.m. (15). As previously mentioned, the sleep duration reported for children in Europe ranged between 9.5 h in Estonia and 11.2 h in Belgium (8). In comparison to data from a recent study of 572 children in India (7), where their sleep duration was 8 ± 1.18 h, the sleep duration in Greece was found to be longer. In a previous study from China, the sleep duration ranged between 8.9 ± 1 and 9.9 ± 1 h in urban and rural areas, respectively (16), while in Saudi Arabia, the total sleep time of elementary school children was 8.4 ± 1.1 on weekdays (17).

The optimal sleep duration for children has been previously questioned (18). However, the U.S. National Sleep Foundation has published specific recommendations for sleep duration according to a child's age, namely 10–13 h for preschoolers, 9–11 h for school-age children, while, for teenagers, the recommended duration is between 8 and 10 h (19). A decrease of 0.75 min/year in children's sleep duration over the past century was reported in a meta-analysis covering 690,747 children from 20 countries (20). In a multi-center study assessing the sleep duration of pre-school and primary school children in eight European countries (8), it was also demonstrated that age progression led to an average decrease by 5.5 min/year in sleep duration, while none of the examined social determinants (i.e., lifestyle factors, parental educational level) were associated with the observed reduction, a result not in accordance with our findings, where a higher educational level of the mother was associated with later sleep onset time.

In our study population, sleep quality in general was reported to be good, and sleep disturbances were mentioned in younger children and in children of less-educated mothers. Reported disturbances mainly refer to the presence of snoring, the prevalence of which was significantly higher (26.1%) than in previous reports. Indeed, in a review and meta-analysis comprising 48 worldwide studies, the estimated prevalence of snoring in childhood was 7.45% (95% CI 5.75–9.61%) (21). In accordance with this, a recent study from Turkey, a country neighboring Greece, estimated the prevalence of primary snoring at 7.2% among 5200 school children (22), while in a recent report from India, it was 11.2% (23). Periodic limb movement was reported by 14.1% of participants, a percentage slightly higher than that reported by Crabtree et al. (24), who estimated its prevalence at 11.9% in a community survey. In other studies, different prevalences were reported, ranging between 5.6 and 26% (25–27).

Another finding of our study was the effect of the educational background of the mother on the sleep characteristics of the children. Less educated mothers reported a sleep disturbance in their children more often. This can be attributed to the fact that they tend to overrate the presence of randomly occurring symptoms. In addition, children of better-educated women tend to go to sleep later at night, contrarily to a report from Saudi Arabia, where children of better-educated mothers went to sleep earlier (17). Most probably, cultural differences play a role in this discrepancy. A possible explanation in our case is that either mothers or children try to extend the time of being together, since better-educated women are usually engaged in more demanding jobs out of the home, while less educated mothers, especially in rural areas, usually stay at home and spend more time with their children.

A limitation of this study is that children with diagnosed chronic health conditions were not included. Nevertheless, the purpose of this study was to examine sleep problems in otherwise healthy children and in relation to various socio-demographic factors. An additional limitation is that factors like excessive noise in the environment, sleeping with a pet in the room, or habits, like computer or other devices use, which are known to be associated with shorter sleep duration or higher prevalence of sleep disturbances (11, 28, 29) were not examined. However, as our results show, among the studied subgroups, no difference was observed in sleep initiation time, a factor which could be attributed to the use of an electronic device prior to sleep; on the contrary, the observed differences were in the time children were awakened.

## Conclusions

In conclusion, in this culturally diverse region of south-eastern Europe, the sleep of children is certainly affected by age progression, but socio-economic factors, like the educational background of the mother, or cultural factors, like belonging in a minority group, may also define its specific characteristics.

## Data Availability Statement

The datasets generated for this study are available on request to the corresponding author.

## Ethics Statement

The studies involving human participants were reviewed and approved by the Medical School, Democritus University of Thrace (1/7-10-2014) and the Institutional Review Board of the University General Hospital of Alexandroupolis, Greece. Written informed consent to participate in this study was provided by the participants' legal guardian/next of kin.

## Author Contributions

DC and PS: conceptualization. EN: methodology, software, validation, formal analysis, and visualization. MK: investigation. AK: resources. DC: data curation, supervision, and project administration. All coauthors: writing—original draft preparation, writing—review, and editing.

### Conflict of Interest

The authors declare that the research was conducted in the absence of any commercial or financial relationships that could be construed as a potential conflict of interest.
